# Asymmetric RNA Distribution among Cells and Their Secreted Exosomes: Biomedical Meaning and Considerations on Diagnostic Applications

**DOI:** 10.3389/fmolb.2017.00066

**Published:** 2017-10-04

**Authors:** Marco Ragusa, Cristina Barbagallo, Matilde Cirnigliaro, Rosalia Battaglia, Duilia Brex, Angela Caponnetto, Davide Barbagallo, Cinzia Di Pietro, Michele Purrello

**Affiliations:** ^1^BioMolecular, Genome and Complex Systems BioMedicine Unit, Section of Biology and Genetics G Sichel, Department of BioMedical Sciences and Biotechnology, University of Catania, Catania, Italy; ^2^IRCCS Associazione Oasi Maria S.S., Institute for Research on Mental Retardation and Brain Aging, Troina, Italy

**Keywords:** exosomes, RNAs, RNA sorting, biomarkers, cancer, asymmetric molecular distribution

## Abstract

Over the past few years, exosomes and their RNA cargo have been extensively studied because of the fascinating biological roles they play in cell-to-cell communication, including the signal exchange among cancer, stromal, and immune cells, leading to modifications of tumor microenvironment. RNAs, especially miRNAs, stored within exosomes, seem to be among the main determinants of such signaling: their sorting into exosomes appears to be cell-specific and related to cellular physiopathology. Accordingly, the identification of exosomal miRNAs in body fluids from pathological patients has become one of the most promising activity in the field of biomarker discovery. Several analyses on the qualitative and quantitative distribution of RNAs between cells and their secreted exosomes have given rise to questions on whether and how accurately exosomal RNAs would represent the transcriptomic snapshot of the physiological and pathological status of secreting cells. Although the exact molecular mechanisms of sorting remain quite elusive, many papers have reported an evident asymmetric quantitative distribution of RNAs between source cells and their exosomes. This phenomenon could depend both on passive and active sorting mechanisms related to: (a) RNA turnover; (b) maintaining the cytoplasmic miRNA:target equilibrium; (c) removal of RNAs not critical or even detrimental for normal or diseased cells. These observations represent very critical issues in the exploitation of exosomal miRNAs as cancer biomarkers. In this review, we will discuss how much the exosomal and corresponding donor cell transcriptomes match each other, to better understand the actual reliability of exosomal RNA molecules as pathological biomarkers reflecting a diseased *status* of the cells.

## Introduction

The complex functional coordination among different cell types, tissues and organs in Metazoans is made possible thanks to cell-to-cell communication (Gerdes and Pepperkok, 2013). Cells are able to communicate by soluble factors (e.g., hormones, cytokines), adhesion molecules mediating cell-to-cell interactions, and specialized cellular structures (e.g., cytonemes, nanotubules), which connect neighboring cells and enable the transfer of surface components and cytoplasmic molecules (Majka et al., 2001; Rustom et al., 2004; Sherer and Mothes, 2008). Cell communication is critical also in specific pathological processes, including tumorigenesis. Indeed, cancer cells need to cross-talk to each other, normal cells, and immune system to survive, proliferate, and metastasize. Communication among tumor and stromal cells leads to modifications of tumor microenvironment favoring tumor growth, survival, immune-escape, and invasion (Brucher and Jamall, 2014). Moreover, long-distance communication with stroma located at distant non-cancerous sites facilitates the formation of metastatic niches and promotes metastatic processes (Ungefroren et al., 2011). Although the molecular mechanisms promoting cell-to-cell communication have not been fully understood yet, recent studies have shown that cells may also communicate by secreting and exchanging small membranous particles named extracellular vesicles (EV). Initially, EVs were considered to be cellular debris released by cells, following cell damage or dynamic plasma membrane turnover (Siekevitz, 1972). Inconsistent with this initial hypothesis, in 1977 it has been shown that EVs can also be the result of a specific and active cellular process and that they may carry functional membrane enzymes (De Broe et al., 1977). Cells can secrete different types of EVs that are classified according to their subcellular origin (Colombo et al., 2014). EVs may come from the cell plasma membrane as shedding vesicles by direct budding of plasmatic membrane. Such EVs display a wide range of diameter sizes (100–1,000 nm) and are generally known as microvesicles; depending on their cellular origin, EVs also have been named ectosomes (from polymorphonuclear leukocytes), microparticles (from platelets), or argosomes (secreted during the morphogenesis of multicellular organisms from basolateral epithelial membranes; George et al., 1982; Hess et al., 1999; Greco et al., 2001). Another source of EVs is represented by apoptotic bodies, which originate from apoptotic cells and range from 1 to 5 μm in diameter (Hristov et al., 2004). Otherwise, EVs may arise from multivesicular bodies from the endosomal compartment (EC): after its fusion with the plasma membrane, EC releases intraluminal vesicles into the extracellular space as exosomes (Thery et al., 2002). Exosomes are vesicles spanning from 30 to 100 nm in diameter, which are enriched in endosomal molecules as tetraspanin proteins CD9, CD63, CD81, and CD82 (Lotvall et al., 2014). Therefore, EVs circulating in body fluids are an extremely heterogeneous population, which differ in cellular origin, size, and surface molecules, and are secreted by nearly all cell types in both physiological and pathological conditions. Over the past 10 years, researchers have mainly focused on unveiling the functional role and pathological involvement of EVs (above all, exosomes), especially after the discovery by Valadi et al. that exosomes carry fully functional mRNAs and miRNAs able to modify the physiological state of recipient cells (Valadi et al., 2007). The RNA contents of exosomes is heterogeneous and may depend on different biomolecular factors, including the specific cellular *status*. Some miRNAs are highly expressed in exosomes, while others are barely present in them; moreover, some miRNAs are enriched only in exosomes secreted by distinct cell types (Pigati et al., 2010; Nolte-'T Hoen et al., 2012; Villarroya-Beltri et al., 2013). All these clues suggest that RNA molecules are not randomly loaded into exosomes, but that rather a machinery actively regulating the sorting of specific sets of RNAs into exosomes does exist. Similar to other molecular *apparati*, this molecular machinery may be functionally perturbed by drugs, diseases and infections (Purrello et al., 1998; Di Pietro et al., 2009).

Exosomes carry distinctive sets of mRNAs, rRNAs, miRNAs, other small non-coding RNAs (ncRNAs; e.g., piRNAs, snRNAs, snoRNAs, scaRNAs, Y-RNAs), and long non-coding RNAs (Li M. et al., 2014; Van Balkom et al., 2015). The exosomal cargo is critical to determine the outcome of cell communication (Mittelbrunn et al., 2011; Hergenreider et al., 2012; Halkein et al., 2013). As reported above, cancer-derived exosomes are able to modulate the immune response against the tumor, and promote angiogenesis, invasion, and metastasis (Milane et al., 2015; Silva and Melo, 2015). Much evidence has suggested that miRNA signatures of tumor-derived exosomes may be used as potential circulating biomarkers for the diagnosis of several types of cancers (Munson and Shukla, 2015; Soung et al., 2017). Moreover, circulating exosomal miRNAs have also been reported as candidate biomarkers for pregnancy disorders, liver damage and inflammation, cardiovascular diseases, and neurodegeneration (Cosme et al., 2013; Masyuk et al., 2013; Tsochandaridis et al., 2015; Soria et al., 2017). Intriguingly, some disease-related exosomal miRNAs mirror pathway dysfunctions of their source cells (Haug et al., 2015; Reclusa et al., 2016; Zhong et al., 2016). The discovery of exosomal miRNAs circulating in body fluids and their potential exploitation as non-invasive disease biomarkers have given rise to the following question (Ma R. et al., 2012; Wang J. et al., 2016): how exactly exosomal RNAs represent an accurate transcriptomic snapshot of the physiological and pathological status of their source cells? To date, many studies have dealt with such question directly or indirectly, but results from them are conflicting and not conclusive. In this review, we will summarize the most critical issues on the sorting of RNA molecules into exosomes and we will discuss how much the exosomal and corresponding donor cell transcriptomes match each other, in order to better understand the actual reliability of exosomal RNA molecules as pathological biomarkers reflecting the diseased *status* of cells.

## Mechanisms of molecular sorting into exosomes

Different mechanisms for sorting molecules into exosomes have been described, although the precise molecular signaling controlling them are unsatisfactorily known (Villarroya-Beltri et al., 2014). Endosomal Sorting Complexes Required for Transport (ESCRT) controls the sorting of ubiquitinated proteins into Intraluminal Vesicles (ILVs) through a molecular cascade involving several ESCRT sub-complexes (Henne et al., 2011). Specifically, ESCRT-0 binds ubiquitinated proteins and is associated to the endosomal compartment thanks to its interaction with phosphatidylinositol 3-phosphate (PI3P). ESCRT-0 recruits ESCRT-I, which in turn recruits ESCRT-II proteins, which lastly activate the ESCRT-III machinery. Snf7 protein (an ESCRT-III component) forms oligomeric assemblies inducing vesicle budding and recruits the adaptor protein ALG-2-Interacting Protein X (Alix) to stabilize the ESCRT-III complex (Henne et al., 2011). ESCRT-independent mechanisms of sorting into exosomes have been also reported (Stuffers et al., 2009). Proteolipid-positive exosomes are enriched in cholesterol and ceramide and their secretion is closely related to the production of ceramide by neutral sphingomyelinase 2 (nSMase2; Trajkovic et al., 2008). Indeed, nSMase2 controls the secretion of Aβ-peptide-exosomes in neurons, whereas the ceramide induces a curvature of the endosomal membranes and the coalescence of microdomains, leading to the budding of intraluminal vesicles (Yuyama et al., 2012). Another process independent from the ESCRT machinery could be regulated by tetraspanins, integral membrane proteins that are highly abundant on the exosome surface. Tetraspanins are able to form intra-membrane tetraspanin-enriched domains by interacting with other membrane proteins and lipids (Escola et al., 1998; Yanez-Mo et al., 2009): for instance, CD81 structurally organizes the membranes in microdomains, while CD63 regulates the loading of LMP1 protein into exosomes and PMEL into intraluminal vesicles during melanogenesis (Van Niel et al., 2011; Verweij et al., 2011; Perez-Hernandez et al., 2013). The specific mechanisms of RNA sorting into exosomes are still poorly characterized and represent a matter of debate. The sorting of RNA molecules within mammalian cells appears to be independent of ESCRT and dependent on ceramide (Kosaka et al., 2010). It has been proposed that RNA loading into exosomes occurs before the budding process, when RNA molecules bind to raft-like regions of multivesicular body membranes creating intraluminal vesicles through inward budding (Janas and Janas, 2011; Janas et al., 2012). RNA binding to membranes is determined by hydrophobic modifications, lipid structures, and sphingosine at physiological concentration in rafted membranes (Janas et al., 2015). It has also been reported that specific nucleotide sequences show enhanced affinity to phospholipid bilayers (Khvorova et al., 1999; Vlassov et al., 2001; Janas and Yarus, 2003; Janas et al., 2004). Bolukbasi et al. suggested that the loading of mRNAs into exosomes could be mediated by a specific zipcode-like sequence, present within the 3′UTR of mRNAs that are enriched in exosomes, and by the presence of binding sites for miRNAs that are highly expressed in source cells (Bolukbasi et al., 2012). Computational analysis of over-represented motifs in the sequence of miRNAs that are enriched in exosomes, along with mutagenesis experiments, led to the identification of specific nucleotide motifs (named EXOmotifs) that may regulate the loading of miRNAs into exosomes. EXOmotifs are recognized and bound by the heterogeneous ribonucleoprotein A2B1 (hnRNPA2B1). Specifically, the sumoylated form of hnRNPA2B1 performs miRNA sorting into exosomes (Villarroya-Beltri et al., 2013). Previously, hnRNPA2B1had been reported to be involved in the intracellular transport of specific mRNAs in neurons and HIV genomic RNAs to packaging sites (Munro et al., 1999; Levesque et al., 2006). Intriguingly, Signal Recognition Particle RNAs (SRP-RNAs), also present within exosomes, are bound by Signal Recognition Particle (SRP) proteins through a GGAG tetraloop, which shares the same sequence of one of the two EXOmotifs previously identified (Wild et al., 2001). Recently, a MALDI-TOF/TOF mass spectrometry analysis of proteins specifically binding to exosome-enriched miRNAs in an *in vitro* hepatocyte model revealed a new molecular player in the sorting of miRNAs into exosomes: synaptotagmin-binding cytoplasmic RNA-interacting protein (SYNCRIP; Santangelo et al., 2016). Knockdown of SYNCRIP impaired the exosomal loading of specific exosome-enriched miRNAs, whereas RNA immunoprecipitation showed that SYNCRIP binds directly to some EXO-miRNAs thanks to a short common sequence (the hEXO motif) that is shared by 60% of exosome-enriched miRNAs (Santangelo et al., 2016). Similarly to hnRNPA2B1, SYNCRIP is also sumoylated, but the hEXO motif which it binds to is different from the EXOmotifs bound by hnRNPA2B1. This suggests that the exosome sorting machinery might involve different ribonucleoproteins, each controlling the exosomal loading of a specific set of miRNAs. Bang et al. found that about one-quarter of miRNAs found in fibroblast-derived exosomes is represented by miRNAs derived from the passenger strand (also named *star miRNAs*), which usually undergo intracellular degradation (Bang et al., 2014). This suggests the existence of a preferential transport mechanism of *star miRNAs* into exosomes. Intriguingly, Squadrito et al. showed that miRNA distribution in macrophages and their exosomes depends on the cellular levels of their target transcripts (Squadrito et al., 2014). They found a negative correlation between miRNA:target interactions in cells and miRNA enrichment in exosomes. Physiological or artificial overexpression of either specific miRNAs or their target mRNAs promoted a bidirectional miRNA redistribution from the cell cytoplasm/P bodies to the multivesicular bodies and, accordingly, a controlled miRNA sorting into exosomes (Squadrito et al., 2014). In other words, when the mRNA target of a specific miRNA is abundant in the cytoplasm, that miRNA needs to stay in the cytoplasm in order to bind to it, and, as a consequence, its amount decreases within exosomes. On the contrary, a miRNA, more abundant in the cytoplasm than its target, will have an increased level also in the exosomes. These findings would suggest that secretion of miRNAs into exosomes is a mechanism through which cells, at least in part, can arrange miRNAs in excess respect to their targets in order to adjust the physiological miRNA:mRNA homeostasis (Squadrito et al., 2014). Altogether, these observations would suggest that RNA sorting into exosomes is the complex result of: (a) active mechanisms of molecular loading, based on specific sequence features of RNA molecules; (b) partially passive processes, which depend on the availability of RNA molecules free and unrestrained from functional binding mechanisms inside the cells. However, these considerations could be challenged by a study showing that miRNAs, even the most abundant, are present at far less than one copy per exosome, suggesting that the most of exosomes circulating in biological fluids are almost empty (Chevillet et al., 2014). These results do not underplay the role of exosomes in cell-to-cell communication, but rather provide some interesting scenarios on exosome sorting and uptake mechanisms. Indeed, the authors propose two models: (1) a low-occupancy/low-miRNA concentration model, in which a sub-population of exosomes carries a low amount of miRNAs; or (2) a low-occupancy/high-miRNA concentration model, in which rare exosomes inside the same nanovesicle population carry many copies of a given miRNA. This latter appears to be the most consistent with exosome-mediated communication, if exosome uptake is a selective and infrequent event. However, low-occupancy/low-miRNA concentration model could be considered valid if cellular uptake of exosomes is rapid, and therefore miRNAs can accumulate within the cell in functionally sufficient quantities. Both these models lead to the hypothesis that an exosome population from the same cell source exhibits a dramatic quantitative heterogeneity of its miRNA cargo (Chevillet et al., 2014). The molecular determinants of such heterogeneity of exosome loading remains still uncharacterized.

## Asymmetric distribution of RNA molecules between source cells and their exosomes

Several papers on *in vitro* cellular models have analyzed the qualitative and quantitative distribution of RNAs between cells and their secreted exosomes, both at steady states and after biological *stimuli*. In one of the earliest reports, Pigati et al. found that nearly 30% of exosomal miRNAs released by normal and malignant mammary epithelial cells does not reflect the cellular profile, suggesting that some miRNAs are selectively retained or released (Pigati et al., 2010). Nevertheless, they observed that about 66% of the released miRNAs had an amount that closely reflected the corresponding abundance inside the cells. These findings would suggest that the majority of miRNAs is passively secreted through exosomes depending on miRNA mass. Intriguingly, the authors also reported that miRNAs with well-characterized roles in mammary biology tend to be selectively retained by cells and nearly absent in the extracellular population. This category of miRNAs included: miR-196a-1, which is overexpressed in breast cancer cells; miR-210, a hypoxia sensor with prognostic value in breast cancer; other miRNAs associated with metastasis (e.g., miR-148a, miR-335, miR-373, and miR-520c). In flat opposition to this group of retained miRNAs, the authors found miR-451 to be one of the most disproportionately exported miRNAs: more than 90% of it was secreted into the extracellular space (Pigati et al., 2010). MiR-451 functions as a tumor suppressor in breast cancer: its active removal from cells might represent a *convenient* mechanism to promote cancer progression (Liu et al., 2015). Furthermore, the excessive release of miR-451 might also explain the interstitial accumulation of miR-451 reported in breast tumors (Sempere et al., 2007). This asymmetric distribution of miR-451 has also been reported in another study on normal and malignant mammary epithelial cells and their exosomes, in which the authors observed the selective encapsulation of miRNAs inside the exosomes and characterized different miRNA profiles between the exosomes secreted by cancer cells and those produced by normal cells (Hannafon et al., 2016). Among the exosome-enriched miRNAs, those that were more abundant in exosomes from the breast cancer cell lines MCF7 and MDA-MB-231 than in exosomes from the normal cell line MCF10A were miR-21, miR-122, miR-451, and miR-1246 (Hannafon et al., 2016). Interestingly, miR-21 and miR-1246 were also highly expressed in plasma exosomes from patients affected by breast cancer. Although both miRNAs are highly abundant in breast cancer exosomes, they are also in large measure retained inside the cells because of their oncogenic role in breast cancer. On the contrary, miR-122 and miR-451 are downregulated in breast cancer cells and have tumor suppressive properties (Wang et al., 2012; Liu et al., 2015). The observations from these two studies on mammary epithelial cells further strengthen the idea that miRNAs secreted via exosomes may represent a mixture of: (a) highly expressed cellular miRNAs, which passively pass through the endosomes for an *osmotic-like effect*; (b) selectively secreted miRNAs, whose function inside the cytoplasm could be unnecessary or unfavorable for the cells in certain time frames and in specific physiological or pathological conditions (Pigati et al., 2010; Hannafon et al., 2016). This hypothesis has also been proved by studies on other cell types. Unequal distribution of RNAs between endothelial cells and their exosomes has also been reported (Van Balkom et al., 2015). More specifically, the authors observed a partially overlapping distribution of small RNAs in exosomes and corresponding donor cells. As a matter of fact, the most abundant miRNAs within the cells matched the most numerous ones in exosomes. However, about half of the identified miRNAs and 5p-, 3p-, and stem-loop fragments were differentially distributed. Just as an example: (1) miR-30d, miR-30e, miR-92b, and miR-125a were among the most abundant miRNAs in cells, but they were barely present in exosomes; (2) miR-25, miR-27a, miR-186, and miR-4485 were the most abundant miRNAs in exosomes, but were scarcely present inside the cells (Van Balkom et al., 2015). Interestingly, the authors focused their attention also on other RNA molecules than miRNAs: they observed that mRNA, lncRNA, vRNA, mtRNA, and yRNA fragments were more enriched in exosomes than in cells. These data on RNA molecule fragments in exosomes would suggest a link between RNA turnover and exosome biogenesis: endothelial endosomes could selectively sequester cytoplasmic RNA-degrading machineries and release degraded RNAs via exosomes (Van Balkom et al., 2015). In a previous work from our group, we have analyzed the miRNA transcriptome of two different colorectal carcinoma (CRC) cell lines and their secreted exosomes before and after treatment with Cetuximab, a monoclonal antibody that binds and blocks the Epidermal Growth Factor Receptor (EGFR; Ragusa et al., 2014, 2015b). About 90% of cellular miRNAs were also present inside the exosomes for both cell lines. However, in contrast with such a qualitative miRNA overlapping, we found a strong quantitative asymmetry of miRNAs between secreted exosomes and their source cells. Some miRNAs were mainly released into exosomes (e.g., miR-127, miR-136^*^, miR-144^*^, miR-432, miR-433, miR-487b, and miR-495, in Caco-2 exosomes; miR-136^*^, miR-223^*^, miR-380-5p, miR-432, and miR-672, in HCT-116 exosomes). Exosomes from both cell lines were enriched in miRNAs with a potential tumor suppressive role in CRC or with immunosuppressive properties (e.g., miR-142-5p, miR-150, miR-223, and miR-433; Ragusa et al., 2014). This finding was consistent with our functional data showing a decreased proliferation of Caco-2 cells after transfection with HCT-116-derived exosomes and *vice versa* (Ragusa et al., 2014). This anticancer effect of tumor-derived exosomes on tumor cells had been previously reported for pancreatic cancer cells (Ristorcelli et al., 2008). Asymmetric distribution of miRNA contents in exosomes and their source cells was highly accentuated by Cetuximab treatment in Caco-2 (Cetuximab sensitive), but less pronounced in HCT-116 (Cetuximab unresponsive). The sets of differentially expressed miRNAs in exosomes and their source cells were different for these two CRC cell lines. However and interestingly, for both of them the exosomal miRNAs that were most dysregulated after treatment were involved in many cancer-related functions, as oncomirs or tumor suppressors, and in inflammatory processes (Ragusa et al., 2014). We concluded that cellular miRNAs, both at steady state and after pharmacological treatment, are selectively released into the tumor microenvironment through exosomes in order to: (a) throw functionally disadvantageous miRNAs out of the cells, or (b) favorably influence the immune system response (Ragusa et al., 2014). We obtained similar data on the asymmetric distribution of retained cellular miRNAs and secreted exosomal ones in an *in vitro* model of non-alcoholic fatty liver disease (NAFLD) and non-alcoholic steatohepatitis (NASH; Di Mauro et al., 2016). Interestingly, in HepG2 cells at steady state we found that: (1) 104 miRNAs were specifically expressed within the cells; (2) 20 miRNAs were exclusively secreted in the culture medium; (3) 284 miRNAs were both present inside the cells and secreted in the medium. In the same cell line analyzed after lipotoxic and non-lipotoxic *stimuli* we found quantitative, but not qualitative, alterations for many secreted miRNAs, and just few and relatively minor changes for those intracellular (Di Mauro et al., 2016). The majority of extracellular miRNAs were involved in inflammatory-related pathways. This finding led us to propose the following hypothesis: in response to lipogenic *stimuli*, some miRNAs could be selectively secreted to either act in an endocrine manner and determine a cross-talk among NAFLD-associated inflamed tissues or act in a paracrine/autocrine manner and regulate inflammation in hepatocytes or innate immune liver cells (Di Mauro et al., 2016). Our results suggest that the innate disequilibrium between retained and secreted miRNAs could be perturbed by biochemical *stimuli* (Ragusa et al., 2014; Di Mauro et al., 2016). A similar rearrangement of the cellular environment by exosomes has been observed in a study on HIV infection (Aqil et al., 2014). In this work, it has been demonstrated that transfection of U937 cells with the HIV Nef protein (a multifunctional virulence factor) leads to the selective secretion through exosomes of 47 miRNAs and retention of 2 miRNAs in Nef-expressing cells. Intriguingly, exosomes secreted in response to the intracellular upregulation of Nef were enriched in miRNAs that target proinflammatory cytokines and can potentially attack HIV-1 (Aqil et al., 2014). In this way, Nef expression would reduce the intracellular levels of miRNAs responsible for innate immune responses and targeting viral transcripts by throwing them out of the cell via the exosomes. This mechanism could potentially be used by the virus to modify the host cell environment in favor of its replication and dissemination. In general, miRNA sorting into exosomes has been demonstrated to be largely influenced by virus infection. Just to cite a different and peculiar example, lymphoma cell lines infected by Kaposi sarcoma-associated herpes virus (KSHV) have been found to produce exosomes that contained about 48% of miRNAs of viral origin (Hoshina et al., 2016). This phenomenon is due to the expression of viral small RNAs in the host cells: some of these viral miRNAs may possess EXOmotifs, like CCCT or CCCG, that influence the exosome loading of the host cells. Even more interesting is the finding that exosomes from virus-infected cells more frequently contained non-exact mature miRNAs (i.e., mutated miRNAs) than the corresponding infected source cells (Hoshina et al., 2016). This observation would suggest the presence of a mechanism that preferentially sorts non-exact mature miRNAs to the exosomes. Moreover, this would inspire the idea that exosomes might have the function of removing the mutated mature miRNAs from cells and, accordingly, concentrating wild-type and functional miRNAs into the cytoplasm. Otherwise, the delivery of non-exact miRNAs to target cells via exosomes could have other unexpected and unknown roles and functions (Hoshina et al., 2016). All these data on the asymmetrical distribution of RNAs between exosomes and source cells lead us to some critical considerations on the biological meaning of RNAs released by exosomes and to potential general sorting criteria. The first evident issue is that cells tend to retain and accumulate into the cytoplasm RNAs that exert critical functions for the cell itself in both physiological and pathological conditions. We note that a *normal* epithelial cell expresses and retains RNAs involved in its *correct* specific physiology and homeostasis. Nevertheless, when the same cell undergoes cancer transformation, it will tend to retain RNAs that promote cell growth and inhibit cell death. Cells preferentially hold back RNAs that are advantageous for cell functioning in specific conditions (Kanlikilicer et al., 2016). This consideration leads us to a second question: which RNAs do cells secrete via exosomes? Different from RNAs retained within the cytoplasm, those encapsulated in exosomes are preferentially RNAs that could be judged to be not critical for the appropriate cell performance or detrimental in normal or diseased conditions. Moreover, also mutated miRNAs and other species of degraded RNAs are largely abundant in exosomes (Van Balkom et al., 2015; Hoshina et al., 2016). The expulsion of these *useless*, dysfunctional, or potentially deleterious RNAs out of the cells would represent a convenient cellular process to accelerate the turnover of *non-effective RNAs*. Intriguingly, when secreted exosomes are captured by recipient cells, this apparent *waste* RNA cargo can in some cases be functionally effective: the main effect reported for some pathological conditions is the modification of the cell environment to favor cancer dissemination or virus replication (Meckes et al., 2010; Pegtel et al., 2010; Neviani and Fabbri, 2015). However, stating that cells retain into the cytoplasm functionally useful RNAs and throw out useless or disadvantageous ones via exosomes would be a largely misleading and inappropriate over-simplification. Indeed, miRNAs harboring EXOmotifs or hEXO motifs are selectively transported by specific RNA-binding proteins into exosomes, regardless of their amount or mutated form (Villarroya-Beltri et al., 2013; Santangelo et al., 2016). Moreover, the most abundant cytoplasmic miRNAs are passively incorporated into the exosomes, especially when they exceed the amount of their mRNA targets and, accordingly, are not engaged in Ago2 binding (Squadrito et al., 2014). In this way, these miRNAs become unconstrained from any functional commitment in the cytoplasm and can be passively stored inside the exosomes. This observation could appropriately explain the presence of oncomir-enriched exosomes in the blood of cancer patients, as discussed in the next paragraph.

## Exosomal miRNAs appear to be promising biomarkers in cancer patients, but caveats are needed

**Figure 1:**
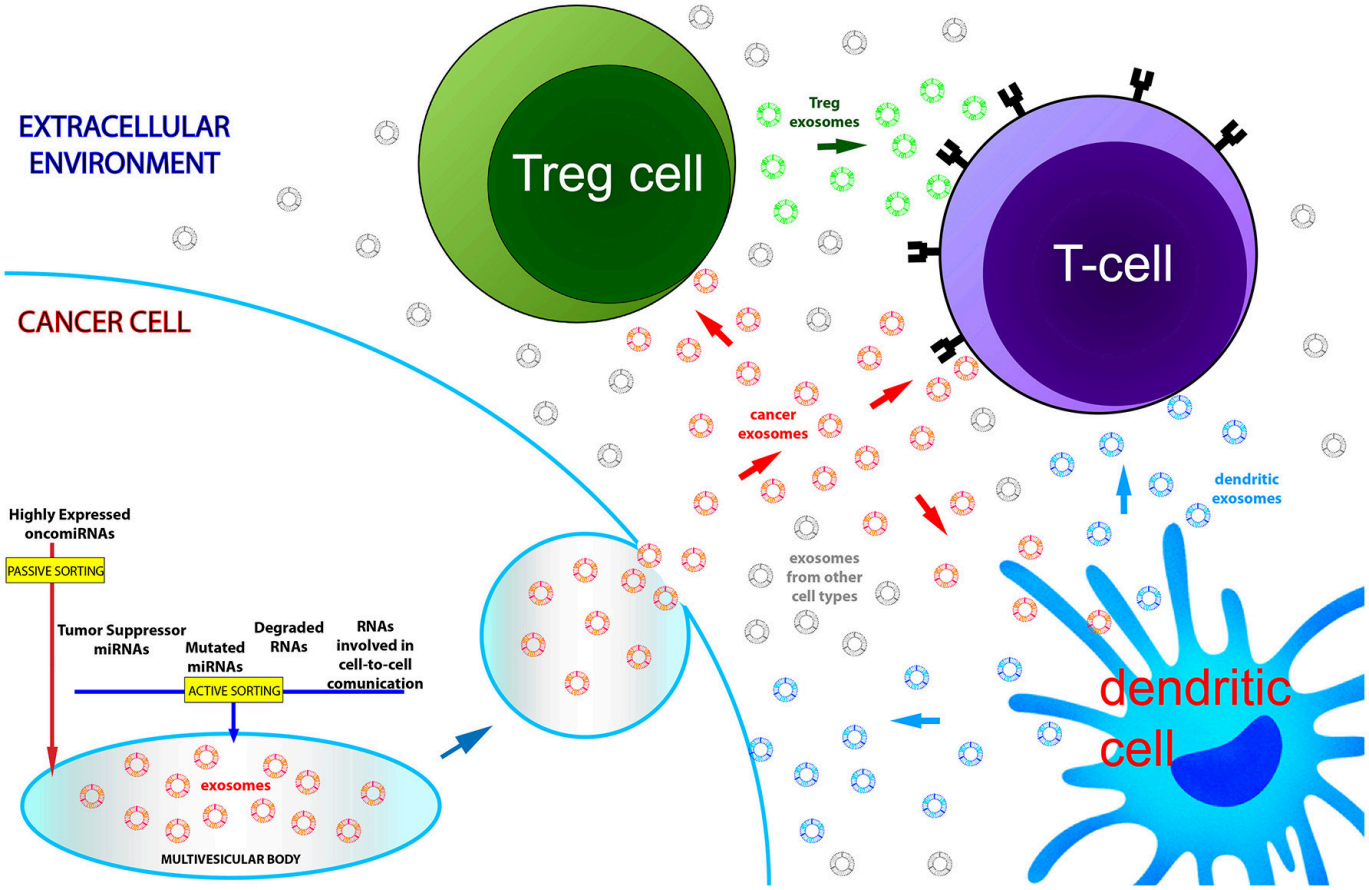
Exosomal RNA dysregulation partially reflects transcriptomic alterations of parental cancer cells. Active and passive sorting mechanisms are responsible for the native RNA quantitative asymmetry existing between cells and exosomes. In blood of cancer patients the nanovesicle population is the complex outcome of exosome production by multiple cell types: cancer cells and immune cells, through exosomal secretion, regulate their molecular homeostasis and communicate with each other during cancer development and progression. For these reasons, exosomal RNAs recovered from the systemic circulation only partially mirror the transcriptome of tumor cells.

Our knowledge on the exact relationship between the profile of exosomal miRNAs circulating in body fluids and the pathological condition of cancer patients is admittedly incomplete: this notwithstanding, the potential of exosomal miRNAs and other ncRNAs as cancer biomarkers is very promising. First of all, miRNAs encapsulated in exosomes are very stable because they are protected against RNase degradation by the lipid bilayer (Valadi et al., 2007). Moreover, exosomal miRNA expression may be easily analyzed by using whatever liquid of our body, even though the most of studies have focused on the exosomes purified from systemic blood circulation (Keller et al., 2011; Revenfeld et al., 2014; Boukouris and Mathivanan, 2015). As stated in the previous paragraph, RNA molecules stored in exosomes represent the molecular outcome of both active and passive sorting mechanisms, which seem to be cell-specific. Exosomes circulating in the blood are a heterogeneous population of nanovesicles derived from a *plethora* of different cell types, but principally coming from blood cells (Chen et al., 2008; Hunter et al., 2008; Pritchard et al., 2012b; Zhou et al., 2017). Accordingly, exosomal miRNAs purified from serum or plasma are a mixture of molecules of different cellular origins and with different roles in extracellular communication. Given these premises, it is hardly to be expected that in the whole population of secreted exosomes, dysfunctional exosomes derived from diseased cells may possess an RNA cargo able to markedly modify the relative amounts of circulating miRNAs. Nevertheless, multiple evidence shows that: (1) exosomal miRNAs from serum or plasma of cancer patients are quantitatively altered; (2) interestingly, some of these dysregulations mirror those detected in source tumor cells (Table 1). A fitting example is the one about miR-21. MiR-21 is among the most commonly upregulated miRNAs in cancer: its genetic locus at 17q23 is amplified in many solid tumors and its expression is stimulated by a variety of cancer-associated phenomena, such as inflammation and hypoxia (Griffin et al., 1988; Wu et al., 2001; Loffler et al., 2007; Fujita et al., 2008; Ribas et al., 2009). Notably, several studies have reported increased levels of miR-21 in both serum and plasma exosomes from patients affected by tumors showing a cellular miR-21 upregulation (Skog et al., 2008; Taylor and Gercel-Taylor, 2008; Que et al., 2013; Ogata-Kawata et al., 2014; Hannafon et al., 2016; Lai et al., 2017; Tsukamoto et al., 2017). Taylor et al. showed that the levels of 8 miRNAs (miR-21, miR-141, miR-200a, miR-200c, miR-200b, miR-203, miR-205, and miR-214) were increased in exosomes from serum of ovarian cancer patients, as well as in tumor tissues from the same patients (Iorio et al., 2007; Nam et al., 2008; Taylor and Gercel-Taylor, 2008; Niu et al., 2015; Azizmohammadi et al., 2016; Xiaohong et al., 2016; Li J. et al., 2017; Wei et al., 2017). Liu et al. observed elevated levels of exosomal miR-23b-3p, miR-10b-5p, and miR-21-5p in plasma of non-small-cell lung cancer (NSCLC) patients and their association with poor overall patient survival (Liu et al., 2017). Interestingly, these three miRNAs have been reported to be also overexpressed in NSCLC cells and furthermore their intracellular expression has been found to be associated with poor prognosis (Begum et al., 2015; Huang et al., 2015; Tian et al., 2016; Xue et al., 2016; Li C. et al., 2017). In a previous work of our group, we showed the constant and consistent upregulation of miR-146a in: (1) vitreous humor, (2) vitreal exosomes, (3) serum, (4) serum exosomes, and (5) cancerous tissues of uveal melanoma patients (Ragusa et al., 2015a). We suggested that miR-146a might be released by melanoma cells inside the ocular chamber and then conveyed to the systemic circulation through tumor blood vessels. Specifically, we observed this fixed expression trend among tissues and body fluids only for miR-146a: this would suggest the existence of specific mechanisms of retention, secretion, and filtering of exosomal miRNAs through the cellular and extracellular compartments (Ragusa et al., 2015a). In contrast with these examples of exosomal miRNA alterations that match the corresponding cellular ones in cancer patients, there are other reports in which the miRNA expression trend in blood exosomes was inconsistent with or even the opposite to the one observed in the corresponding cancer cells (Table 1). Profiling of serum exosomal miRNAs in primary CRC patients showed the upregulation of let-7a, miR-1229, miR-1246, miR-150, miR-21, miR-223, and miR-23a (Ogata-Kawata et al., 2014). It is interesting to note that the overexpression of exosomal miR-23a, miR-223, and miR-1246 conformed with their upregulation reported in CRC tissues and linked to their oncogenic role (Yong et al., 2013, 2014; Della Vittoria Scarpati et al., 2014; Li Z. W. et al., 2014; Zhang et al., 2014; Neerincx et al., 2015; Wang S. et al., 2016; Wang F.F. et al., 2017). On the contrary, augmented serum levels of let-7a and miR-150 were inconsistent with their documented downregulation and tumor suppressive property in CRC (Ma Y. et al., 2012; Li et al., 2016). Recently, another study on serum exosomes from CRC patients reported the upregulation of miR-486-5p and miR-3180-5p, and the downregulation of miR-548c-5p, miR-638, miR-5787, miR-6869-5p, miR-8075 (Yan et al., 2017). Among these differentially expressed miRNAs, miR-638 was the most relevant concerning the clinics: its downregulation has been previously reported to be related to invasion and mesenchymal-like transition in CRC cells (Ma et al., 2014). This is not the case for miR-486-5p: its exosomal upregulation was diametrically opposed to its marked downregulation in CRC tissues, previously reported by Liu et al. (2016). For all the other dysregulated exosomal miRNAs identified in this study the corresponding alterations in CRC tissues have not been reported yet. Wang et al. found miR-125a-3p and miR-320c to be significantly upregulated in plasma exosomes from patients with early stage CRC (Wang J. et al., 2017): there is no experimental evidence of miR-125a-3p alteration in CRC, while miR-320c has been reported to be frequently downregulated in CRC tissues together with the other members of miR-320 family (Tadano et al., 2016; Vishnubalaji et al., 2016). Interestingly, the upregulation of miR-320 was also detected in serum exosomes from Glioblastoma Multiforme (GBM) patients (Manterola et al., 2014), in contrast with miR-320 decreased levels reported in GBM tissues (Guo et al., 2014). Taken together, these data on exosomes circulating in the blood of cancer patients would be an *in vivo* indirect proof that exosomes secreted by cancer cells might carry a cargo of RNA molecules that does not exactly reflect the cytoplasmic RNA alterations of their source cells. This could be considered inessential from a diagnostic point of view. For instance, the upregulation of exosomal miR-21 and miR-320 in serum of CRC patients could represent for clinicians a very useful evidence to diagnose CRC in a non-invasive manner, regardless of miR-21 overexpression and miR-320 downregulation described in CRC source cells (Slaby et al., 2007; Vishnubalaji et al., 2016). However, many works on the differential expression of exosomal miRNAs in cancer have attempted to computationally reconstruct miRNA-based networks in order to infer pathways that might be involved in a specific tumor (Eldh et al., 2014; Alhasan et al., 2016). This approach could be considered misleading: a part of altered circulating miRNAs in body fluids of cancer patients has a relative expression that is largely different from the one in the corresponding diseased tissues. The tumor suppressor miRNA let-7a inhibits tumor cell growth and metastasis in CRC and its levels are significantly decreased in CRC tissues and cell lines (Li et al., 2016); nevertheless, its exosomal levels in serum of CRC patients are increased (Ogata-Kawata et al., 2014). This apparent incongruity might be the result of a strategy adopted by the cancer to remove from cells miRNAs that are disadvantageous for viability and dissemination of CRC. This *scenario* is made even trickier by the presence in circulation of exosomes from other cellular sources that could contribute to the cancer-related miRNA dysregulation reported in literature (Figure 1). The presence of exosome populations of different origin could dilute the actual expression alterations deriving from cancer cells. However, it is worth mentioning that exosome concentrations are increased in cancer patients compared to normal controls (Taylor and Gercel-Taylor, 2008; Logozzi et al., 2009; Caivano et al., 2015; Alegre et al., 2016), suggesting the hypothesis that cancer cells release huge quantity of exosomes due to their intrinsic cancer-related mutations (Yu et al., 2006). Moreover, exosomes coming from immune cells could have an important role in changing the expression of miRNAs within the whole exosomal population. Cancer development elicits an immune response, triggered by tumor antigens distinguishing the cancerous cells from the other non-cancerous ones (Grivennikov et al., 2010). On one hand, tumor-infiltrating lymphocytes act by slowing or arresting the development of the tumor. On the other hand, cancer cells are able to favorably influence the tumor microenvironment by inducing signaling cascades, leading to immune-suppression and thus facilitating evasion of immune surveillance and cancer dissemination (Gardner and Ruffell, 2016; Jang et al., 2017). This *fight* between tumor and immune system is also fought by means of exosome secretion by both cancer cells and regulatory immune-cells (Bobrie and Thery, 2013; Greening et al., 2015). Indeed, tumor-derived exosomes directly suppress the anti-tumor responses of cytotoxic T lymphocytes and NK-cells, and also induce the generation, expansion, and suppressive function of T regulatory cells (Treg). Okoye et al. demonstrated that Treg cell-derived exosomes are able to transfer both *in vitro* and *in vivo* a specific set of miRNAs to T cells (e.g., miR-155, let-7b, and let-7d), thus suppressing T helper cell proliferation and interferon-γ production (Okoye et al., 2014). On the contrary, several reports demonstrated that activated dendritic cells secrete exosomes and induce a T-cell-mediated anti-tumor immune response (Zitvogel et al., 1998; Viaud et al., 2010; Tian and Li, 2017). These observations lead us to consider that part of dysregulated exosomal miRNAs in blood of cancer patients could derive from different activated regulatory immune cells and act by suppressing or activating the immune system. In agreement with such hypothesis of the over-representation of cancer- and immune-derived exosomes in the systemic circulation of cancer patients, it should not be surprising that a large number of exosomal miRNAs proposed as cancer biomarkers also has a critical functional role in the differentiation and activation of immune cells (e.g., miR-10b, miR-17, miR-20a, miR-146a, miR-150, miR-181a, miR-223; Table 1; Paladini et al., 2016). This could explain why in some cases exosomal miRNAs differentially expressed in the blood of cancer patients have no links to altered cytoplasmic miRNAs of the native tumor. There is a final technical warning that may be worth mentioning to fully understand the effectiveness of exosome as cancer biomarkers. One of potential pitfalls of studies on exosome functions and their molecular cargo is the lack of standard methods to obtain highly pure exosome populations. Several methods are reported in scientific literature, including ultracentrifugation, density gradient centrifugation, chromatography, filtration, polymer-based precipitation, and immunoaffinity (Taylor and Shah, 2015). It has been demonstrated that these methods could lead to co-isolate contaminating non-exosomal material or to the loss of exosomes due to damaged membrane integrity, thus, resulting in significant artifacts of recovery, quality and molecular content of exosomes (Whiteside, 2017). Just as an example: ultracentrifugation is the “gold standard” exosome isolation method, but the type, quantity and quality of the vesicles isolated is extremely sensitive to parameters, including *g* force, rotor type, angle of rotor sedimentation, radius of centrifugal force, solution viscosity, and vesicle density (Taylor and Shah, 2015). Needless to say, accounting for, controlling and standardizing all of these parameters is quite impossible. Van Deun et al. performed a comparative study of 4 exosome isolation protocols (i.e., ultracentrifugation, OptiPrep density gradient centrifugation, and two commercial polymer-based precipitation methods) to evaluate their reliability for downstream molecular analyses (Van Deun et al., 2014). The four methods provided different qualitative and quantitative results, but OptiPrep density gradient ultracentrifugation outperformed the other 3 methods and revealed a unique and reproducible mRNA profile. In a similar work, Tauro et al. compared ultracentrifugation, density gradient centrifugation and immunoaffinity to purify exosomes from biological fluids and conditioned media from *in vitro* cell cultures (Tauro et al., 2012). Their proteomic analyses revealed that immunoaffinity capture was the most effective method to isolate exosomes. Indeed, the immunoaffinity capture is considered the better alternative for exosome isolation, because there is no damage to the nanovesicle structure and the loss of exosomes is negligible. All these considerations strongly suggest that the choice of specific isolation method severely affects the purity and quality of exosomes and, accordingly, the reliability of molecular profiles of their RNA content. Moreover, also the use of disparate methods to profile RNA expression from exosomes could add further bias to final data and cause a scanty reproducibility among different studies (Git et al., 2010; Chugh and Dittmer, 2012; Pritchard et al., 2012a; Moldovan et al., 2014).

**Table 1:** Expression of miRNA cancer biomarkers in exosomes and their relative tumor tissues.

**miRNAs**	**Cancer**	**Exosome origin**	**Expression within exosomes**	**PMID EX**	**Expression within cells**	**PMID CELL**
let-7a	Colon cancer	Serum	Up	24705249	Down	27498032
let-7a	Pancreatic ductal adenocarcinoma	Plasma	Down	28232049	Down	19323605
miR-10b	Pancreatic ductal adenocarcinoma	Plasma	Up	28232049	Up	22018284
miR-10b-5p	Non-small-cell lung cancer	Plasma	Up	28055956	Up	25988292
miR-17-5p	Pancreatic adenocarcinoma	Serum	Up	24007214	Up	27400681
miR-20a	Pancreatic ductal adenocarcinoma	Plasma	Up	28232049	Down	25485236
miR-21	Breast cancer	Plasma	Up	27608715	Up	16103053, 18812439, 26549725
miR-21	Colon cancer	Serum; plasma	Up	24705249; 28376502	Up	18196926, 22844381, 28376502
miR-21	Glioblastoma	Serum	Up	19011622	Up	21895872
miR-21	Glioma	Cerebrospinal fluids	Up	26284486	Up	16024602, 24326156
miR-21	Ovarian cancer	Serum	Up	18589210	Up	17875710, 18451233
miR-21	Pancreatic adenocarcinoma	Serum; plasma	Up	24007214; 28232049	Up	20093556, 22850622, 28239461
miR-21	Uveal melanoma	Vitreus	Up	25951497	Up	25951497
miR-21-5p	Non-small-cell lung cancer	Plasma	Up	28055956	Up	26453197, 27811366, 28445945
miR-21-5p	Prostate cancer	Urine	Up	26417675	Up	16461460, 27040772
miR-23a	Colon cancer	Serum	Up	24705249	Up	23758639, 24992592
miR-23b-3p	Non-small-cell lung cancer	Plasma	Up	28055956	Up	26314549
miR-30c	Pancreatic ductal adenocarcinoma	Plasma	Up	28232049	/	/
miR-34a	Uveal melanoma	Vitreus	Up	25951497	Up	25951497
miR-106b	Pancreatic ductal adenocarcinoma	Plasma	Up	28232049	/	/
miR-122	Pancreatic ductal adenocarcinoma	Plasma	Down	28232049	Down	22850622, 28239461
miR-125a-3p	Colon cancer	Plasma	Up	28646161	/	/
miR-140-3p	Chronic myeloid leukemia	Serum	Up	28197964	/	/
miR-141	Ovarian cancer	Serum	Up	18589210	Up	17875710, 18451233
miR-141-5p	Prostate cancer	Urine	Up	26417675	/	/
miR-146a	Uveal melanoma	Serum	Up	25951497	Up	25951497
miR-146a	Uveal melanoma	Vitreus	Up	25951497	Up	25951497
miR-150	Colon cancer	Serum	Up	24705249	Down	22052060
miR-181a	Pancreatic ductal adenocarcinoma	Plasma	Up	28232049	Up	17473300
miR-200a	Ovarian cancer	Serum	Up	18589210	Up	17875710, 18451233
miR-200b	Ovarian cancer	Serum	Up	18589210	Up	17875710, 18451233
miR-200c	Ovarian cancer	Serum	Up	18589210	Up	17875710, 18451233
miR-203	Ovarian cancer	Serum	Up	18589210	Up	27347348, 27655286
miR-205	Ovarian cancer	Serum	Up	18589210	Up	26275944, 28145479, 28476165
miR-214	Ovarian cancer	Serum	Up	18589210	Up; down	17875710; 18451233
miR-223	Colon cancer	Serum	Up	24705249	Up	24819398, 25270282, 27916606
miR-320	Glioblastoma multiforme	Serum	Up	24435880	Down	25117070
miR-320c	Colon cancer	Plasma	Up	28646161	Down	27119506, 27559432
miR-486-5p	Colorectal cancer	Serum	Up	28636562	Down	27284245
miR-548c-5p	Colorectal cancer	Serum	Down	28636562	/	/
miR-574-3p	Glioblastoma multiforme	Serum	Up	24435880	/	/
miR-574-3p	Prostate cancer	Urine	Up	26417675	Down	23554959
miR-638	Colorectal cancer	Serum	Down	28636562	Down	24885288
miR-1229	Colon cancer	Serum	Up	24705249	/	/
miR-1246	Breast cancer	Plasma	Up	27608715	/	/
miR-1246	Colon cancer	Serum	Up	24705249	Up	25143946, 26436952, 26573378
miR-3180-5p	Colorectal cancer	Serum	Up	28636562	/	/
miR-6869-5p	Colorectal cancer	Serum	Down	28636562	/	/
miR-8075	Colorectal cancer	Serum	Down	28636562	/	/
miR-5787	Colorectal cancer	Serum	Down	28636562	/	/

*This table reports the trend of expression of exosomal miRNA biomarkers in cancer and in tumor parental cells. PMID EX, Pubmed IDs of papers about exosomal miRNAs; PMID CELL, Pubmed IDs of papers about cancer cellular miRNAs. Slash: no data available*.

## Conclusions

The key concepts reviewed in this paper may be summarized in the three following points. (1) RNA molecules secreted via exosomes are encapsulated thanks to both active, scarcely characterized, cell-specific mechanisms of sorting and also passive processes depending on the amount of cytoplasmic RNAs. The biological meaning of the active processes remains largely elusive: however, it appears to be related to the maintenance of the correct molecular equilibrium between RNA molecules that are biologically important, ineffective, or potentially deleterious for the cell in a certain physiological or pathological condition. Moreover, secreted RNAs might have a role in the homeostasis of the extracellular microenvironment. (2) This co-occurrence of both active and passive transport processes leads to a partial match between exosome and source cell transcriptomes, as observed in the *in vitro* models reviewed in this paper. (3) Differentially expressed exosomal miRNAs in the circulation of cancer patients only partly reflect miRNA dysregulations found in corresponding cancer tissues and, as a matter of fact, divergent expression trends between exosomes and native tumors have been documented for many miRNAs. These differences could be the assorted result of the asymmetric distribution of miRNAs between exosomes and cells, but also the confounding effect of the presence in the blood of exosomes from different cellular origins. Specifically, the regulatory cells of the immune system can induce the activation or suppression of T cells in the tumor microenvironment by producing regulatory exosomes and, thus, they significantly influence the total relative expression of circulating miRNAs.

Finally, exosomal miRNAs could be considered as good biomarkers in cancer, even if no standard reproducible method to isolate them has been proposed. Nevertheless, they do not represent an accurate reflection of miRNA intracellular expression in the diseased cells, but they are the complex outcome of a mixed exosome production from multiple cell types, which through exosomal secretion regulate their molecular homeostasis and communicate with each other during cancer development and progression.

## Author contributions

All authors contributed to the writing of the paper, performed a critical revision of the review and approved the final manuscript.

### Conflict of interest statement

The authors declares that the research was conducted in the absence of any commercial or financial relationships that could be construed as a potential conflict of interest.
